# C-reactive protein ratio response patterns in pediatric sepsis: a cohort study - preliminary results

**DOI:** 10.1186/2197-425X-3-S1-A787

**Published:** 2015-10-01

**Authors:** V Soares Lanziotti, P Póvoa, L Pulcheri, PZ Meirelles, G Guimarães, AS Mendes, MO Ribeiro, M Soares, JIF Salluh

**Affiliations:** Federal University of Rio de Janeiro, Pediatric Intensive Care Unit, Rio de Janeiro, Brazil; D'Or Institute for Research and Education, Critical Care Research, Rio de Janeiro, Brazil; New University of Lisbon, Nova Medical School - UCIP - Hospital São Francisco Xavier, Lisbon, Portugal; Rio's D'Or Hospital, Pediatric Intensive Care Unit, Rio de Janeiro, Brazil

## Introduction

Despite numerous advances in treatment and supportive care over the last years, sepsis remains the most important risk factor for mortality in infants and children in the world, according to the World Health Organization. Besides, clinical judgment is insufficient to an early identification of outcomes in septic children.

## Objectives

The aim of the present study is to evaluate the patterns of C-reactive Protein (CRP) ratio response to antibiotic therapy during the first week in the Pediatric Intensive Care Unit (PICU) in patients admitted with sepsis.

## Methods

This is a multicenter prospective descriptive cohort of children (1 month-old to 18 year old) admitted at PICUs of 3 tertiary hospitals in Rio de Janeiro, Brazil, with sepsis diagnosis (< 72 hours). CRP was sampled every other day from D0 to D6 of PICU stay. CRP-ratio was calculated in relation to D0 CRP concentration. Patients were classified according to an individual pattern of CRP-ratio response with the following criteria: fast response - when D4 CRP was less than or equal to 0.4 of D0 CRP concentration; slow response - when D4 CRP was > 0.4 and D6 less than or equal to 0.8 of D0 CRP concentration (continuous and slow response); nonresponse - when D6 CRP was > 0.8 of D0 CRP concentration; biphasic response - characterized by an initial CRP decrease to levels < 0.8 of the D0 CRP followed by a secondary rise > 0.8. Comparison between ICU survivors and non-survivors was performed.

## Results

Preliminary results of this cohort included 57 patients (age median: 1.6; 56,1% male); the most frequent sites of infection were lung 54,4%, central nervous system 14%, abdominal 7%. Out of the 57 patients, 50 could be classified according to a predefined pattern: N=15 fast response, N=23 slow response, N=5 nonresponse and N=7 biphasic response. The ICU mortality rate was significantly different according to the patterns of CRP-ratio response: fast response 6,7%, slow response 4,3%, nonresponse 40% and biphasic response 42,9% (p < 0.001). The time dependent analysis of CRP-ratio course of the different patterns was significantly different (p < 0.001).

## Conclusions

Preliminary results of this pediatric sepsis cohort suggest that sequential evaluation of CRP-ratio is useful in the early identification of patients with poor outcome. The evaluation of CRP-ratio pattern of response to antibiotics during the first week of therapy could be useful in the recognition of the individual clinical evolution, influencing clinical decision-making process at bedside.

